# The Role of NAD^+^ and NAD^+^-Boosting Therapies in Inflammatory Response by IL-13

**DOI:** 10.3390/ph17020226

**Published:** 2024-02-08

**Authors:** Anton D. Pugel, Alyssa M. Schoenfeld, Sara Z. Alsaifi, Jocelyn R. Holmes, Brad E. Morrison

**Affiliations:** 1 Biomolecular Ph.D. Program, Boise State University, Boise, ID 83725, USA; antonpugel@boisestate.edu; 2 Department of Biological Sciences, Boise State University, Boise, ID 83725, USA; alyssaschoenfeld@u.boisestate.edu (A.M.S.); saraalsaifi@u.boisestate.edu (S.Z.A.); jocelynholmes@u.boisestate.edu (J.R.H.)

**Keywords:** NAD^+^, IL-13, allergies, nicotinamide riboside, nicotinamide mononucleotide

## Abstract

The essential role of nicotinamide adenine dinucleotide^+^ (NAD^+^) in redox reactions during oxidative respiration is well known, yet the coenzyme and regulator functions of NAD^+^ in diverse and important processes are still being discovered. Maintaining NAD^+^ levels through diet is essential for health. In fact, the United States requires supplementation of the NAD^+^ precursor niacin into the food chain for these reasons. A large body of research also indicates that elevating NAD^+^ levels is beneficial for numerous conditions, including cancer, cardiovascular health, inflammatory response, and longevity. Consequently, strategies have been created to elevate NAD^+^ levels through dietary supplementation with NAD^+^ precursor compounds. This paper explores current research regarding these therapeutic compounds. It then focuses on the NAD^+^ regulation of IL-13 signaling, which is a research area garnering little attention. IL-13 is a critical regulator of allergic response and is associated with Parkinson’s disease and cancer. Evidence supporting the notion that increasing NAD^+^ levels might reduce IL-13 signal-induced inflammatory response is presented. The assessment is concluded with an examination of reports involving popular precursor compounds that boost NAD^+^ and their associations with IL-13 signaling in the context of offering a means for safely and effectively reducing inflammatory response by IL-13.

## 1. Introduction

The most prominent case of disease caused by altered nicotinamide adenine dinucleotide^+^ (NAD^+^) levels is that of pellagra—a disease characterized by dementia, dermatitis, diarrhea, and possibly even death caused by NAD^+^ deficiency arising from a poor diet [1]. The development of pellagra was determined to be directly related to low niacin levels (a known precursor of NAD^+^) [2]. Pellagra is now quite rare in the United States due to the fortification of foods with niacin; however, it is still a major health issue for impoverished nations. Following its discovery, systemic applications of NAD^+^ became of primary research interest due to its role in metabolic functioning, cellular signaling, and DNA repair [3]. It has been established that low NAD^+^ levels are associated with a wide range of diseases, both inherited and acquired. In fact, alterations in the biosynthesis of NAD^+^ are thought to play a role in the development of prevalent conditions, including metabolic disorders, neurological diseases, congenital defects, and cardiovascular complications [4].

As a substrate of sirtuins, NAD^+^ plays a significant role in metabolic functions and disorders. Phosphorylated NAD^+^ has been shown to be depressed in mouse models of pre-diabetes and type II diabetes. Increasing NAD^+^ levels resulted in improved non-fasting glucose levels and glucose tolerance, normalized HbA1c, and reduced weight gain in these mice [5]. Additionally, the complications of diabetes resulting from poor vascular function—such as retinopathy, hypertension, and myocardial infarction—are due to low mobilization of endothelial progenitor cells caused by NAD^+^ deficiency in the nicotinamide phosphoribosyltransferase (NAMPT)-NAD^+^ pathway [6]. NAD^+^ also has myocardial protective properties, preventing apoptosis and stimulating autophagic flux in cardiac myocytes [7]. 

**Figure 1:**
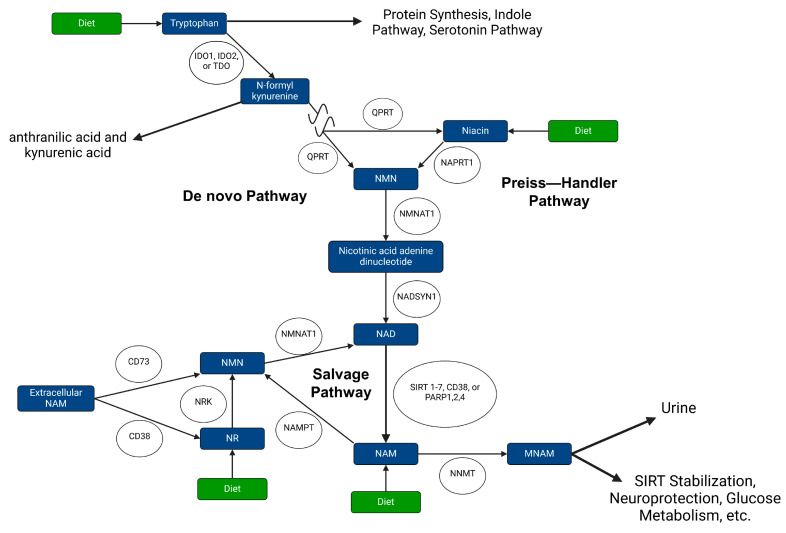
NAD^+^ homeostasis. NAD^+^ levels are tightly regulated via de novo, Preiss–Handler, and salvage pathways. Abbreviations: nicotinamide (NAM), nicotinamide mononucleotide (NMN), nicotinamide riboside (NR), 1-methyl-NAM (MNAM), N-methyltransferase (NNMT), indoleamine 2,3-dioxygenase 1 (IDO1), indoleamine 2,3-dioxygenase 2 (IDO2), or tryptophan 2,3-dioxygenase 1 (TDO), nicotinamide phosphoribosyltransferase (NAMPT), NAD Synthetase 1 (NADSYN1), nicotinate phosphoribosyltransferase (NAPRT1), quinolinate phosphoribosyltransferase (QPRT), nicotinamide nucleotide adenylyltransferase 1 (NMNAT1), Nik related kinase (NRK), poly(ADP-ribose) polymerase (PARP), sirtuin (SIRT). Figure created with BioRender.

Loss of NAD^+^ homeostasis is often the driving force behind many diseases. NAD^+^ homeostasis is maintained through different systems working simultaneously through the metabolism of NAD^+^ and its synthesis from de novo, Preiss–Handler, and salvage pathways (Figure 1). Synthesis of NAD^+^ comes from many precursors such as tryptophan through the de novo pathway; niacin through the Preiss–Handler pathway; and nicotinamide (NAM), nicotinamide mononucleotide (NMN), and nicotinamide riboside (NR) in the salvage pathway [8,9,10]. Through the de novo NAD^+^ pathway, which is part of the kynurenine pathway, tryptophan is converted to N-formyl kynurenine by one of indoleamine 2,3-dioxygenase 1 (IDO1), indoleamine 2,3-dioxygenase 2 (IDO2), or tryptophan 2,3-dioxygenase 1 (TDO). From there, further modifications are made to N-formyl kynurenine until it is converted into NAM or niacin by QPRT. The Preiss–Handler pathway reaches this point with the conversion of niacin to NAM by NAPRT. From here, NAM is converted to nicotinic acid adenine dinucleotide by NMNAT. Nicotinic acid adenine dinucleotide is then converted to NAD^+^ by Nadsyn1. Homeostatic control over the de novo and Preiss–Handler pathways is achieved through its components being directed to other uses in the cell. For example, in addition to being an amino acid, tryptophan is a precursor to the kynurenine pathway, indole pathway, and serotonin pathway [11]. Within the kynurenine pathway, many compounds can branch off into other products besides NAD^+^. This includes N-formyl kynurenine being a precursor to anthranilic acid and kynurenic acid.

NAD^+^ is primarily synthesized through the salvage pathway, which occurs in most tissues, while the other two pathways are predominantly carried out in hepatocytes, the kidney, and macrophages [12,13,14]. The salvage pathway is active when enzymatic activity using NAD^+^ results in the production of NAM and/or ADPR. NAD^+^ is metabolized by a plethora of enzymes in various locations. This includes CD38, which converts NAD^+^ into cyclic ADP-ribose (ADPR), with NAM released [15]. CD38 is an important contributor to controlling NAD^+^ levels, with mice deficient in CD38 having significantly increased NAD^+^ levels [16]. SIRT1-7 utilize NAD^+^ to deacetylate different proteins, including histones, resulting in nicotinamide and acetyl-ADPR [17,18,19]. A buildup of nicotinamide has been shown to have an inhibitory effect on SIRT1 [20]. PARP1, 2, and 4 utilize NAD^+^ to add poly-ADPR chains to proteins as a signaling mechanism for DNA repair, and from this process a NAM is left over [21,22].

Not all nicotinamide that is created from these various enzymatic processes is able to be salvaged. The enzyme responsible for reducing the amount of salvageable NAM is nicotinamide N-methyltransferase (NNMT), which methylates NAM into 1-methyl-NAM (MNAM). This methylation marks the NAM as waste and MNAM is typically excreted from the body in urine [23]. It was initially thought that MNAM played no other biological role, but current studies show that it plays some role in the metabolism of glucose and in neuroprotection, with many more roles being researched [24,25]. NNMT upregulation is related to many diseases, including PD, Alzheimer’s disease, and many cancers [26,27,28,29,30]. NNMT contributes to chemoresistance in breast cancer cell lines through MNAM’s ability to stabilize SIRT1 [30]. This was demonstrated by overexpression of NNMT in an MDA-MBA-231 cell line, resulting in resistance to apoptosis due to the chemotherapies adriamycin and paclitaxel. Knockout mice for NNMT showed decreased renal fibrosis under a unilateral ureter obstruction model, with increased levels of NAD^+^ [31]. NNMT reduces the available NAM to convert into NAD^+^, as MNAM cannot be converted to NAD^+^. In extreme cases where intracellular NAD^+^ is depleted, such as when there is extreme DNA damage and PARPs utilize too much NAD^+^, the cell induces necrosis [22,32].

Exceptions to the three main NAD^+^ synthesis pathways do exist. One example is CD73 being able to convert NMN into NR and CD38 being able to convert NMN into NAM [33,34]. These enzymes have been shown to supply cancer cells with enough NAD^+^ to overcome cell death due to NAD^+^ depletion through NAMPT inhibition [35]. Inhibition of both NAMPT and CD73 has been shown to reduce cancer cell proliferation [36].

In the context of neurology, maintaining proper NAD^+^ homeostasis is essential for neuronal functioning and can prevent neurodegeneration. In excitotoxic conditions, prolonged stimulation of glutamate receptors results in significant energy depletion, neuronal death, and decreased NAD^+^, rendering impaired function of NAD-dependent enzymes and compromising DNA repair mechanisms as a result [37]. Along with managing excitotoxic damage, NAD^+^ balance is a prominent physiological factor in neurodegenerative diseases. Parkinson’s disease (PD) has been associated with a disruption in the resting metabolic state. NAD^+^ levels of the resting tibialis anterior muscle were found to be reduced in PD subjects and positively correlated to an increase in PD symptoms, such as apathy and REM sleep disruption [38]. In addition, accumulation of amyloid beta plaques throughout the brain is a major histopathology of Alzheimer’s disease. Increasing NAD^+^ levels via NR supplementation reduced amyloid beta concentrations in transgenic mice by increasing the expression of peroxisome proliferator-activated receptor-gamma coactivator 1 (PGC)-1alpha in the brain. Through the control of beta-secretase (BACE1) degradation, PGC-1alpha is able to regulate amyloid beta production [39]. 

NAD^+^ metabolism has been linked to other neurodegenerative diseases (Table 1). In Amyotrophic Lateral Sclerosis (ALS), oxidative stress coupled with mitochondrial homeostasis imbalance contributes to neuronal degradation [40]. There is an association with this disorder and an impairment in the functioning of the de novo synthesis pathway. This has been linked to increased cerebrospinal fluid levels as well as an elevation in serum levels of quinolinic acid, kynurenine, and tryptophan. Additionally, ALS also suggests a decline in NAD^+^ as a result of deficiencies in the NAMPT pathway [40].

Recently, metabolic NAD^+^ pathways have garnered interest for their potential role in Multiple Sclerosis (MS). The precursor nicotinamide can ameliorate MS in an experimental encephalomyelitis animal model [41]. As NAD^+^ can be synthesized from the de novo pathway, vitamins B2 and B6 are necessary and a deficiency may lead to accumulation of KP intermediates. KP pathway damage can perhaps lead to the development of ALS [42]. Based on current research, NAD^+^ biosynthesis pathways appear to have significant impact on neurodegenerative diseases. 

**Table 1:** Gene mutations involved with NAD^+^ pathway impairment that are associated with neurodegeneration.

Affected Gene	NAD^+^ Pathway Role for Gene
PARPs, CD38 [3]	NAD degradation; *PARP1* inhibition enhances mitochondrial content [3]
PARPs [3]	Reduction in cytosolic NAD [3]
NAXD [43]	Detoxification of NAD byproduct [43]
NAXE [43]	Inhibition of cellular NADH dehydrogenases [43]

Disruption in NAD^+^ homeostasis can have potentially severe implications for systemic function and health through congenital mechanisms. A likely cause of disruptions are mutations in NAD^+^-associated proteins. During embryogenesis, mammals synthesize NAD^+^ from dietary L-tryptophan via the kynurenine pathway. Biallelic inactivation variations due to missense and frameshift mutations in KYNU, HAAO, and NADSYN1 disrupt this mechanism, causing malformations of the heart, kidney, vertebrae, and limbs [44]. KYNU encodes an enzyme (pyridoxal-5′-phosphate-dependent kynureninase) that generates precursors of NAD^+^ in the synthesis pathway. Meanwhile, HAAO activates after KYNU and produces quinolinic acid. Participants who had inherited biallelic HAAO variants from asymptomatic parents had a variety of congenital cardiac defects. These included tetralogy of Fallot, Shone syndrome, and hypoplastic left heart. Abnormal digits in the hands and feet as well as shortened limbs were noted. Individuals with biallelic KYNU variants and asymptomatic parents had shortened extremities, absent digits and nails, brachymelia, and variable syndactyly, along with cardiac complications. Both HAAO and KYNU variations resulted in NAD^+^ deficiency [44]. 

Further effects of unbalanced NAD^+^ levels involve inflammatory response conditions. Mouse models of Alzheimer’s disease, characterized by neuroinflammation, exhibited decreased NAD^+^ levels [45]. Interestingly, increasing NAD^+^ levels downregulated the gene expression of inflammatory factors such as IL-1, TNF-alpha, and IL-6 [46]. While low NAD^+^ levels are associated with inflammation, high NAD^+^ production through the salvage pathway also plays a role in the development of certain inflammatory disorders. NAD^+^ production (as opposed to L-tryptophan) through the NAMPT salvage pathway results in the excessive use of PARP1 and leads to significant ATP depletion. The resulting oxidative stress has been reported to play a critical role in inflammatory conditions such as psoriasis [46]. In addition to psoriasis, extracellular NAMPT has been found to be increased in irritable bowel syndrome (IBS) patients. In fact, the presence of NAMPT in serum levels is often related to a worse prognosis. These levels tend to increase in patients who have failed anti-TNF-alpha therapy such as infliximab or adalimumab and tend to decrease in patients who have successfully responded to therapy [47].

In relation to inflammatory disease, NAD^+^ has been shown to interact with and directly affect a specific cytokine signaling pathway. Extracellular NAD^+^ levels and activity have been directly linked to the pro-inflammatory cytokine IL-13, which is a key driver of allergic response. Cyclic adenosine diphosphate ribose (cADPR)—a beta NAD^+^ metabolite—contributes to the elevation of intracellular calcium concentrations in the smooth muscle cells of airways and coronary arteries [48]. Calcium is a secondary messenger for muscular contractions and may play a part in airway constriction. IL-13 upregulates CD38, a protein expressed on the surface of airway smooth muscle cells responsible for the synthesis and degradation of cADPR. As a result of this relationship, increased IL-13 results in increased CD38, cADPR, and, consequently, calcium. This proposed mechanism could be how IL-13 evokes inflammatory airway conditions in concert with extracellular NAD^+^ levels [48].

## 2. Examples of NAD^+^’s Cellular Roles

NAD^+^ and NADH play central roles as coenzymes in redox reactions involved in energy metabolism. NAD^+^ is an electron carrier used to store energy during cellular respiration. NADH is a form of NAD^+^ with a high-energy hydrogen electron bond. NADH is generated by NAD^+^ from catabolizing organic metabolites, primarily sugars, via the Krebs cycle inside the mitochondrial matrix. Once created, NADH donates the high-energy electron to the electron transport chain (ETC), where this addition activates H^+^ ion pumps to transport H^+^ into the mitochondrial membrane space. Consequently, ATP synthase creates ATP from ADP and inorganic P when H^+^ is allowed to traverse back to the mitochondrial matrix. This is the primary mechanism for generating the majority of ATP by human cells during oxidative respiration. 

Outside of its direct energy transportation and capture role, NAD^+^, through interaction as a coenzyme or allosteric factor, can influence a variety of cellular functions that are critical for maintaining metabolic homeostasis, including metabolic pathways, DNA repair, chromatin remodeling, and immune cell function [49]. NAD^+^ and NADH participate in many metabolic pathways, playing a regulatory role in processes such as the citric acid cycle, glucose metabolism, and the oxidation of fatty acids [49,50]. In the citric acid cycle, NADH plays not only an integral role as an electron carrier, but also as an inhibitor, as it can inhibit the enzymes involved in the cycle. Additionally, NAD^+^ is crucial for DNA repair mechanisms, chromatin remodeling, and immune cell function, all of which contribute to maintaining metabolic homeostasis [51]. NAD^+^ interacts with PARP1, a protein involved in base excision repair, linking NAD^+^ to proteins as poly-ADP-ribose as a signal for repair in both the mitochondria and nucleus [21,22]. With high levels of DNA damage, PARP1 can deplete internal NAD^+^ stores and trigger cell death [52]. Therefore, NAD^+^ has a multifaceted role in energy metabolism as well as an effect on numerous important and diverse cellular and organismal functions. Interestingly, the role of extracellular NAD^+^ is not well understood, but mounting evidence suggests that this form of NAD^+^ is a potent regulator of immune response [53]. 

## 3. Extracellular NAD^+^ in Inflammatory Response and IL-13 Signaling

**Figure 2:**
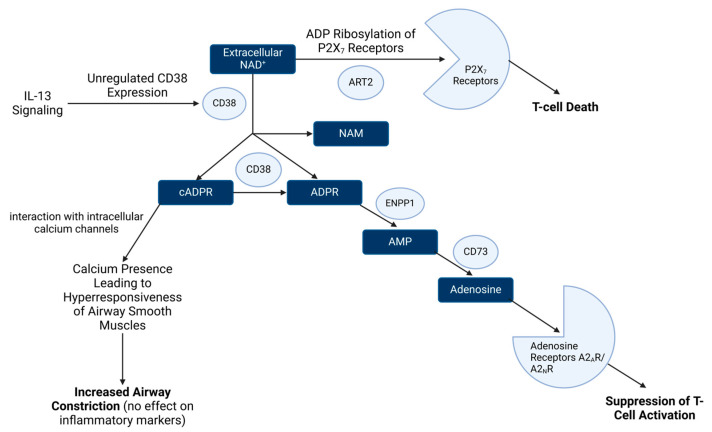
Extracellular NAD^+^ suppresses inflammatory response. NAD^+^ can facilitate repression of inflammatory response through multiple mechanisms. NAD^+^ can induce T cell death via ADP ribosylation of P2X_7_ receptors. In addition, NAD^+^ is catabolized by CD38 into bioactive metabolites that include cADPR. The action of cADPR by smooth muscle cells results in heightened airway hyperresponsiveness without inflammatory marker production. In fact, cADPR drives suppression of inflammatory response through conversion to adenosine by CD73 which inhibits T cell activation via adenosine receptors, making NAD^+^ and cADPR potent anti-inflammatory factors despite the airway constriction induced. Interestingly, IL-13 has been shown to enhance the conversion of NAD^+^ to cADPR through upregulation of CD38 expression. The net effect is that in the presence of elevated extracellular NAD^+^, IL-13 leads to reduced inflammatory response. Figure created with BioRender.

NAD^+^ has been implicated in asthma and allergies primarily through its role as a precursor to a variety of immune cell-modulating compounds. Extracellular NAD^+^ interacts with a wide variety of enzymes in extracellular space and has many functions of its own that include effects on inflammatory response (Figure 2). NAD^+^ can act as a signaling agent through the process of ADP ribosylation, where an ADP-ribose is post-translationally attached to P2X_7_ receptors via mono ADP-ribosyltransferase 2 (ART2), causing intracellular calcium release in T cells and the eventual death of those cells [54]. Additionally, extracellular NAD^+^ has important anti-inflammatory activities through its relationship with CD73. CD73 creates adenosine from several precursors, including NAD-derived cADPR, after NAD^+^ has been processed by CD38 to carry out signaling activities [55]. Adenosine produced by CD73 has potent anti-inflammatory effects, including suppressing T cell function [56,57]. This is evidenced by reports of silencing CD73 in cultured lymphocytes resulting in a pro-inflammatory phenotype [58]. In addition, CD73 knockout mice exhibit increased microglial inflammatory response under lipopolysaccharide-induced inflammation models [59]. Taken together, these results suggest that extracellular NAD^+^ in the context of CD73 signaling is anti-inflammatory. 

Many of the extracellular activities of NAD^+^ are facilitated by the effects of the cell surface enzyme CD38 [60]. CD38 is an ectoenzyme found on immune cells that converts NAD^+^ into cADPR, ADPR, and NAM that leads to immune cell suppression [61]. In fact, there are considerable efforts to target CD38 by antibody therapy to increase inflammatory response in solid tumors for these reasons [62,63,64]. As aging occurs, CD38 accumulates and contributes to the reduction of NAD^+^ levels and an increased presence of its metabolites [61]. These NAD^+^ metabolites also play a role in asthma and allergies through the activity of multiple inflammatory cytokines, including IL-13 [48]. In addition, allergen presence has been reported to cause the release of NAD^+^ into the extracellular space, which can then be utilized by CD38 [65]. Concerning airway diseases, cADPR facilitates the opening of the ryanodine receptors (RyRs) on smooth muscle cells [66]. RyRs are ion channels responsible for the activation and opening of intracellular calcium stores, resulting in the hyperresponsiveness of the airway smooth muscle [67]. This airway hyperresponsiveness increases the ability of smooth muscles surrounding airways to constrict in the presence of agonists, which leads to increased airway closure [48,68]. Additionally, the expression of CD38 has been shown to be increased significantly by IL-13 signaling [69]. It is important to note that IL-13 is a key driver of allergic inflammatory response. This is evidenced by the findings that IL-13 and its receptor IL-13RA1 are required for inflammatory response in a variety of allergy models in mice [70,71,72]. CD38 has an important role in the hyperresponsiveness of airway smooth muscle through the IL-13 signaling pathway, as knockout mice for CD38 have been shown to have significantly decreased airway smooth muscle sensitivity compared to wild type mice in the presence of IL-13, while having no significant effect on inflammation [73]. Therefore, the IL-13 signaling pathway utilizes cADPR to prime the airways to constrict in the presence of allergens but in the presence of high NAD^+^ and/or high CD38 activity, this same IL-13 signaling leads to reduced inflammatory response via CD73-Adenosine-A2_A_R/A2_B_R suppression of T cells. As a consequence, one could theorize that enhancing NAD^+^ levels might counteract the inflammatory milieu during IL-13 release and associated allergic response through these mechanisms. Supporting this notion is a report by Kim et al. [74] where mice that received NAD^+^ precursor compounds (NMN or NR) prior to induction of both systemic and cutaneous anaphylaxis exhibited markedly reduced IgE-mediated anaphylactic responses. However, it is important to note that elevating NAD^+^ levels might also result in enhanced smooth muscle hyperresponsiveness and airway constriction in the context of elevated IL-13 or allergen exposure, which was not assessed in that prior study. A solution to this issue, if shown to be relevant in vivo, might be achieved by enhancing conversion of cADPR to adenosine by CD73, thereby reducing the propensity of airway constriction while still augmenting T cell suppression. 

Reports indicate that NAD^+^ can facilitate anti-inflammatory effects through other cytokines as well. Intracellular NAD^+^ levels impact immune cell function, specifically in CD4^+^ T cells deficient in transcription factor A (Tfam) which regulates expression of mitochondrial DNA. CD4^+^ cells lacking Tfam have impaired mitochondrial function due to an imbalanced NADH/NAD^+^ ratio which ultimately leads to reduced amounts of the IL-10 cytokine known for its immunosuppressive abilities [75]. Similarly, protective microglia secrete anti-inflammatory cytokines such as that of IL-4 and IL-10, both of which have been linked to NAD^+^ levels, as increased levels of NAD^+^ can decrease the effects of neuroinflammation [45]. Therefore, NAD^+^-related declines might impact the anti-inflammatory effects of IL-10 and result in neurodegeneration. 

## 4. Strategies for Increasing NAD^+^ Levels

Increased NAD^+^ levels have been linked to mitochondrial metabolism activation and neuroprotection against age-associated diseases [49]. Consequently, directing NAD^+^ homeostasis has been the subject of pre-clinical studies [76]. The administration of NR, nicotinamide adenine dinucleotide phosphate oxidase (NOX), NMN, or P7C3, an NAMPT activator, has shown promise in increasing NAD^+^ levels [77]. Niacin (vitamin B3) is an NAD^+^ precursor that has previously been marketed to increase NAD^+^ levels; however, niacin is outperformed by other compounds [78]. Additionally, niacin causes redness (flushing) of the skin, which is presumed harmless but is an undesirable side effect. As a result, niacin is not discussed here in detail. 

In the salvage pathway, NAMPT first creates NMN from NAM, and then the conversion of NMN to NAD^+^ takes place. Conversion of NMN to NAM is considered a rate limiting step in NAD^+^ synthesis due to the relatively slow activity by NAMPT. NAMPT is seen as a preferable target in supplementation therapies due to this slow activity [79,80]. While information on the effects of NAMPT supplementation in humans is limited, in mice, administration of P7C3 to mice exhibiting type 2 diabetes increased glucose uptake and promoted pancreatic β cell function. Thus, P7C3-mediated NAMPT activation is critical in ameliorating diabetic skeletal muscle phenotypes in mice, and the NAMPT activator P7C3 is a potential treatment strategy for type 2 diabetes that has yet to be tested in humans [81].

Another target within the salvage pathway is the previously mentioned NNMT, an enzyme highly correlated with PD, Alzheimer’s disease, and many cancers. Targeting NNMT would potentially free up NAM to be converted into NAD^+^. Inhibiting NNMT would also prevent the stabilization of SIRT1 by MNAM. NNMT has been identified in many preliminary studies as a good potential therapeutic target for cancers, chronic kidney disease, and metabolic disorders, such as insulin sensitivity [31,82,83]. Many groups have used computational chemistry to attempt to identify small molecule inhibitors for NNMT [84,85,86]. One group has attempted to identify small molecule inhibitors that are MNAM analogs, with their lead candidate reducing MNAM by 80% at a 2 h mark in mice at 50 mg/kg [84]. Refinements in their understanding of NMNT resulted in a compound that has a strong safety profile and is highly effective at reducing MNAM levels [85]. NNMT is not often targeted for neural disorders as it has been found to be neuroprotective, reducing reactive oxygen species generation and damage to the mitochondria [24,87,88]. One hypothesis is that increased NNMT expression in PD and Alzheimer’s disease is a reaction to the underlying cause rather than a driving force in these diseases.

The enzymatic complex NOX utilizes nicotinamide adenine dinucleotide phosphate (NADP^+^) to produce superoxide anions and other ROS. In pathogenic circumstances, excessive ROS produced by NOX promotes apoptotic cell death. Glucose, through NOX activity, has been shown to facilitate pathogenesis of reperfusion injury in recent studies. Therefore, NOX inhibition could potentially mitigate deleterious impacts of hyperglycemia on stroke [89]. There are no inhibitors today that have demonstrated clinical viability as there is insufficient safety and specificity for human development. NAD^+^ is a precursor to NADH, NADP^+^, and NADPH. NAD^+^ and NADH are converted back and forth to one another by an NAD^+^-dependent dehydrogenase and an NADH-dependent oxidase. NAD^+^ is also converted to NADP^+^ by NAD^+^ kinase [79]. NADPH-oxidase-derived ROS are emerging as key regulators in host immune response as well as neutrophilic inflammation [90].

Three clinical trials addressed the efficacy of oral NMN administration in humans and have found that it is tolerable with minimal effects while modestly increasing NAD^+^ levels. Volunteers received 100, 250, and 500 mg of NMN, with no reported adverse effects [91]. Another study demonstrated that oral administration of 250 mg/day of NMN for 12 weeks is both tolerable and safe in healthy people [91]. Blood examinations were conducted as a baseline, as well as at the fourth, eighth, and twelfth weeks of the trial and in the fourth week after administration was complete. There were few differences between the placebo and NMN groups. Largely, the data support the safety of oral administration of 250 mg/day of NMN for 12 weeks in healthy individuals with small increases in NAD^+^ metabolites [91].

NR is currently the most used over-the-counter supplement to increase NAD^+^ levels in tissue, CSF, whole blood, and peripheral blood mononuclear cells (PBMCs) [92,93,94]. This is due to the growing number of studies linking NR with upregulated genes involved in antioxidant responses, protein degradation, and mitochondrial respiration [93]. NR may also have anti-inflammatory effects through the suppression of cellular levels of inflammatory cytokines, peripherally and in the central nervous system [93]. In the NADPARK clinical trial, it was reported that oral NR therapy boosts NAD^+^ and NAD^+^ metabolite levels in the brain as well as in muscle tissue and PBMCs [93]. Interestingly, this study also reported functional improvement for PD patients receiving oral NR [93]. Today, numerous compounds exist on the market for “NAD^+^ boosting” that utilize oral NR supplementation. Clinical studies have shown that this approach can achieve a 142% increase in NAD^+^ after 8 weeks of daily 1000 mg dosing while being well tolerated [92]. Thus, NR oral supplementation has proven to be a safe and effective means for increasing NAD^+^ levels, which could lead to a plethora of desired effects. 

Whether NAD^+^-enhancing supplement therapy inhibits IL-13 activity is unknown. While no studies were located that directly examine NAD^+^ supplements and the IL-13 pathway activity, there are a growing number of investigations that have reported some findings on IL-13 cytokine expression in this context. Therefore, we surveyed the literature for well-established NAD-boosting compounds and assessed their reported association with IL-13 cytokine or the major pathological outcome of the IL-13 signaling-allergic response (Table 2). Each compound surveyed was found to have some reported association with IL-13 signaling and altered allergic response with the exception of NR, which revealed no reported links to IL-13 signaling. 

## 5. Conclusions

The essential need for NAD^+^ in human health is well established. Once thought of as merely a cellular energy shuttle, research has uncovered many more diverse roles for NAD^+^. These roles are not limited to the cytoplasm and mitoplasm as extracellular NAD^+^ signaling, directly and through its metabolites, is a potent regulator of immune cell function. As a result, considerable efforts are being pursued to inhibit extracellular NAD^+^ signaling in cancer by targeting CD38, thereby enhancing immune response [62,63,64]. Emerging evidence suggests that extracellular NAD^+^ might specifically inhibit IL-13 signaling, although a precise molecular mechanism is unknown. Enhanced IL-13 cytokine signaling is central to allergic response and is implicated in PD and some forms of cancer. Therefore, the inhibition of IL-13 is of great interest in the context of numerous diseases. One potentially safe method for reducing IL-13 signaling might be elevating NAD^+^ levels globally within individuals. Fortunately, given the clinical and pre-clinical evidence supporting the importance of heightened NAD^+^ levels in numerous conditions, there have been successful efforts to elevate NAD^+^ using a myriad of NAD^+^ precursor molecules taken orally. Many of these have been thoroughly examined and exhibit a strong safety profile, while boosting NAD^+^ in select tissues. One therapeutic in particular, NR, has been shown to be safe and highly effective at increasing NAD^+^ levels in clinical trials and many pre-clinical models. This review undertook an examination of the literature for the association between these NAD^+^-boosting compounds with effects on IL-13 and the main pathological outcome of IL-13 signaling, allergic response. There is little information available in the literature regarding this notion. Our findings indicate that more research into NAD^+^-IL-13 signaling and this promising strategy for IL-13-mediated inflammatory response should be further explored. 

## Figures and Tables

**Table 2:** NAD^+^-enhancing supplements and reported effects on IL-13 signaling and allergic response. Summarized PubMed search results using the name of each of the compounds listed in conjunction with the following terms: “IL-13”, “IL-13RA1”, “allergic response”, or “allergies” are shown. Publications that report an effect for an indicated compound on the indicated pathway are shown with a “+” and the associated reference. “N” indicates that no supporting studies were found.

	IL-13 Signaling	Allergic Response
**Niacin**	+ [95]	+ [96]
**Nicotinamide riboside**	N	+ [74,97]
**Nicotinamide mononucleotide**	+ [98]	+ [74]
**NAMPT**	+ [99]	+ [100]
**Nicotinamide**	+ [99]	+ [96]
**Nicotinamide adenine dinucleotide phosphate oxidase**	+ [101]	+ [102]

## Data Availability

No new data were created or analyzed in this study. Data sharing is not applicable to this article.
